# The Impact of Low-Fidelity Three-Dimensional-Printed Models of the Equine Distal Limb and the Canine Forelimb in Teaching Veterinary Anatomy in Practical Classes

**DOI:** 10.3390/ani15101380

**Published:** 2025-05-10

**Authors:** Rebecca Schirone, Maximiliane Schmedding, Janet Weigner, Martin Werner, Giuliano Mario Corte, Jan Peter Ehlers, Luise Grace Klass, Mahtab Bahramsoltani

**Affiliations:** 1 Institute of Veterinary Anatomy, School of Veterinary Medicine, Freie Universität Berlin, Koserstraße 20, 14195 Berlin, Germany; janet.weigner@fu-berlin.de (J.W.); ma.werner@fu-berlin.de (M.W.); grace.klass@fu-berlin.de (L.G.K.); mahtab.bahramsoltani@fu-berlin.de (M.B.); 2 ISME Bern and Avenches, Vetsuisse Faculty, University of Bern, Hochschulstrasse 6, 3012 Bern, Switzerland; maximiliane.schmedding@t-online.de; 3 Institute of Veterinary Anatomy, Vetsuisse Faculty, University of Zurich (UZH), Winterthurerstrasse 260, 8057 Zurich, Switzerland; giuliano.corte@uzh.ch; 4 Didactics and Educational Research in Health Science, Faculty of Health, Witten/Herdecke University, Alfred-Herrhausen-Straße 50, 58455 Witten, Germany; jan.ehlers@uni-wh.de

**Keywords:** veterinary education, experimental study, learning outcomes, student evaluation, formaldehyde-free alternatives, education sciences, musculoskeletal system

## Abstract

Limited cadaver availability and health risks from formaldehyde-treated cadavers have increased the need for alternative learning materials in veterinary anatomy education. Two studies were conducted to investigate whether low-fidelity 3D-printed models provide comparable learning outcomes to real specimens. In study 1, veterinary students were assigned to two groups to study the equine distal limb on a real specimen (*n* = 67) or a 3D model (*n* = 68). In study 2, students were divided into four groups to study the canine forelimb on a real specimen (*n* = 44), a 3D model (*n* = 45), a 3D model followed by a real specimen (*n* = 47), or the reverse combination (*n* = 47). Learning outcomes were measured through a knowledge assessment. Afterwards, learning materials were evaluated by students. Both studies showed significantly higher learning outcomes in the groups learning with real specimens compared to other test groups. While students preferred to start learning with 3D models before switching to real specimens, this method had the lowest learning outcomes. However, students emphasized the value of 3D models for independent learning outside the dissection hall and desired further 3D models of other anatomical specimens. Findings indicated that low-fidelity 3D-printed models cannot replace real specimens but are a useful complementary tool in veterinary anatomy education.

## Simple Summary

Due to limited access to animal cadavers and health concerns associated with cadavers that have been treated with formaldehyde, alternatives are needed for teaching veterinary anatomy. This study tested whether 3D-printed models with simplified representations of anatomical structures help students learn as effectively as with real specimens. In two studies, veterinary students’ knowledge was tested after studying an unfamiliar anatomy topic, using either real specimens, 3D models, or a combination of both, starting with 3D models or real specimens, followed by the other. In both studies, students who used real specimens performed better on knowledge assessments. However, in the subsequent evaluation, many students stated that they prefer to start learning with 3D models followed by the real specimens, although this method had the lowest learning outcomes in the knowledge assessments. Moreover, students found 3D models helpful for learning anatomy regardless of location. They also expressed interest in having additional 3D models of other anatomical specimens. The results suggest that while 3D models cannot fully replace real specimens, they can be a useful addition to veterinary education.

## 1. Introduction

Anatomy is one of the most important foundational subjects in veterinary medical education and provides an essential basis for clinical training [1]. Practical training in veterinary anatomy is usually carried out on cadavers, which creates a number of challenges. A major challenge is the limited availability of anatomical specimens for dissection [2]. These specimens are exclusively obtained from deceased or euthanized animals, which are voluntarily donated by their owners to veterinary anatomy institutes for educational purposes [1]. To extend their usability as teaching materials, formaldehyde in various concentrations is used for different fixation purposes [3]. Although the concentrations are relatively low, formaldehyde was reclassified by the European Union as a “potential carcinogen” in June 2014 [4]. Consequently, strict handling protocols are necessary to minimize potential health and environmental risks associated with formaldehyde exposure [3]. Despite preservation efforts, anatomical specimens remain subject to gradual deterioration over time, leading to structural degradation, loss of key anatomical features, and diminished educational value [5]. Therefore, there is a growing need for alternative approaches to the use of cadavers in veterinary anatomy [1].

An alternative, increasingly applied in clinical teaching, is the use of models on which clinical skills can be simulated. These models provide students with repeated opportunities to practice routine veterinary procedures in a low-pressure setting, reducing the need to use live animals [6,7,8,9]. Students who used the models reported higher confidence in procedure preparation and hand-ties compared to those who did not use them [10]. A distinction is made between low-fidelity models, which offer a simple and reduced representation of an animal’s body or part of a body, and high-fidelity models, which mimic appearance, haptics, and functionality as realistically as possible. Studies show that similar learning outcomes, measured by observing students performing clinical tasks using a standardized checklist, can be achieved with low-fidelity models as with high-fidelity models when learning clinical skills [7,11]. Several 3D-printed, low-fidelity canine models for veterinary clinical skills training were used in a study, focusing on intravenous catheterization, bandaging, and anesthesia practice. Student satisfaction was assessed through a survey, revealing a positive attitude towards the use of these models as clinical simulators [9]. Therefore, low-fidelity models have become a valuable tool in human and veterinary medicine clinical training [9,10,11,12,13,14,15,16].

Low-fidelity models are also used in human anatomical teaching, albeit to a lesser extent than in clinical training [17,18,19,20,21,22,23,24,25,26]. Chan et al. described the use of various low-fidelity models from different human anatomists [17]. Among these, there was a low-fidelity model for learning midgut rotation [23], a model of the digital extensor mechanism designed to illustrate the anatomy and function of the intrinsic muscles [24], a paper model of the muscles [25], and the pipe-cleaner brachial plexus model [26]. Hindmarch et al. evaluated a low-fidelity inguinal canal model [18], while Dixit et al. developed a neuroanatomy low-fidelity model [19]. Only a few studies report the use of low-fidelity models in veterinary anatomical training [2,27,28]. A study from Kinnison et al. tested the use of a haptic simulator to teach bovine abdominal anatomy. Based on a satisfaction survey, the results demonstrate that the haptic simulator provides an effective and engaging approach for teaching bovine abdominal anatomy to large groups of students, reducing the reliance on cadavers and addressing several existing challenges in anatomy education [2]. In another study, 3D-printed models were developed for various structures, including bones, internal organs, and vascular systems, from a range of domestic and exotic animal species. These models were evaluated by comparing them to traditional teaching materials, such as fixed and plastinated specimens, through a student satisfaction survey administered after training sessions. Additionally, a general survey was conducted to gather students’ opinions on traditional teaching methods. Overall, the 3D-printed models were well received by students, although acceptance varied depending on the specific model assessed [27]. The use of 3D-printed canine skulls from three different breeds was evaluated for educational purposes. Both animal science and veterinary students participated by completing a questionnaire regarding their learning experience with the 3D models. The results showed that students from both groups successfully recognized and understood the morphological differences between the skulls, suggesting that 3D-printed models are effective tools for teaching about rare skeletal specimens [28]. Very little is known about learning outcomes when using low-fidelity models in anatomy education. One study in human anatomy assessed learning outcomes using a multiple-choice test. In this study, the intervention group performed better than the control group. However, students in the intervention group received the low-fidelity model in addition to the learning materials that were also available to the control group [18]. Another study evaluated the effectiveness of an otoscopy simulator as a teaching tool to enhance knowledge of middle-ear anatomy and pathology, using a knowledge assessment questionnaire. The questionnaire was administered either at the beginning (control group) or after completion of the simulation (simulation group). Results showed that the simulation group achieved higher scores compared to the control group [21]. Moreover, another study assessed learning outcomes using a pre- and post-test design to compare virtual high-fidelity and low-fidelity liver models, and found no significant differences between the groups [22]. Given the limited research on the use of low-fidelity models in veterinary anatomy education, especially with 3D-printed models, the question arises whether such models could also be beneficial for teaching veterinary anatomy.

This study investigated the use of low-fidelity 3D-printed models compared to real anatomical specimens as learning material in veterinary anatomy classes on the musculoskeletal system. Two hypotheses regarding the effectiveness of 3D-printed low-fidelity models in anatomical education were tested. The first hypothesis proposed that students learning the anatomical structures of the equine distal limb using 3D-printed models would achieve the same learning outcomes as those using real specimens. The second hypothesis suggested that students studying the anatomical structures of the canine forelimb with 3D-printed models would achieve the same learning outcomes as those using real specimens.

## 2. Participants, Materials and Methods

### 2.1. Participants

Data for this study were collected at the Institute of Veterinary Anatomy, School of Veterinary Medicine, Freie Universität Berlin, during practical anatomy lessons on the equine distal limb for second-year veterinary students in June 2024 (study 1) and on the canine forelimb for first-year veterinary students in December 2024 (study 2). The veterinary medicine degree program consists of a total of 11 semesters, of which anatomy is taught in the first four semesters. According to the German Veterinary Licensing Regulations [29], a total of 224 teaching hours of anatomy are required. Each teaching session in anatomy consists of three teaching units, each lasting 45 min. Both present studies were conducted within the framework of one teaching session each and were integrated into the regular teaching schedule. In study 1, the topic of the teaching session was the equine distal limb, including vessels, nerves, tendons, and ligaments. In study 2, the topic of the teaching session was the canine forelimb, including muscles and nerves. One week prior to the study, students were informed about the objectives and the schedule of the study, both verbally and through an information letter. Participation was voluntary, and students provided informed consent by completing and signing a data protection declaration before taking part. They were assured that data collection was anonymous, and they could withdraw from participation at any time without consequences. Additionally, students were instructed not to prepare in advance for the lessons on the equine distal limb (study 1) and the canine forelimb (study 2). The information letter and data protection declaration are provided in Supplement S1.

### 2.2. Learning Materials

In accordance with ethical approval and to prevent increasing students’ workload, anatomical specimens of the musculoskeletal system that are integrated into regular practical anatomy classes were selected for the studies. For study 1, which served as a pilot study, the equine distal limb was selected as a specimen with relatively few anatomical structures to be identified by students. For study 2, the canine forelimb was selected as a specimen with a larger number and complexity of anatomical structures.

#### 2.2.1. Real Anatomical Specimens

In study 1, a total of 32 real anatomical specimens (real specimens) of the equine distal limb were used, consisting of 16 thoracic limbs and 16 pelvic limbs obtained from eight adult horses. The mean length of the equine distal limbs was 48.2 cm (±6.48), with measurements taken from the proximal point of the cannon bone to the distal aspect of the hoof wall.

Frozen equine distal limbs were defrosted cautiously at +4 °C in a cold storage room and then cleaned with a soap solution. Next, the skin was resected, and the remaining limb was fixed in a 30% ethanol–water solution (ethanol dehydrated, denatured with methyl ethyl ketone 1%) (Berkel AHK, Berlin, Germany) using immersion in boxes to preserve the natural mobility and color of the ligaments, as well as clear delineation of ligaments, vessels, and bones. Throughout the preparation, the limbs were repeatedly transferred to a new 30% ethanol solution to preserve the tissue structure and achieve gentle fixation and coloring. Between dissections, the equine distal limbs fixed in ethanol were stored in closed boxes at +4 °C. For the study, 16 distal limbs were dissected to preserve superficial structures, including the annular ligaments, superficial nerves, and vessels (superficial view). In the remaining 16 distal limbs, the palmar/plantar annular ligament and the proximal digital annular ligament were cut laterally to the flexor tendons to allow an unobstructed view of the underlying structures (deep view).

In study 2, a total of 32 real specimens of the canine forelimb were used, consisting of 16 right forelimbs and 16 left forelimbs obtained from 16 adult dogs. The mean length of the canine forelimbs was 50.4 cm (±8.27), with measurements taken from the dorsal margin of the scapula to the coronary border of the third claw.

Frozen canine forelimbs were gently thawed in a cold storage room (+4 °C) and then cleaned using a soap solution. Next, perfusion fixation of the individual limbs was performed manually via the axillary artery using a tied-in button cannula. In some cases, the fixation solution did not reach all muscle groups completely. In such cases, a targeted post-injection was necessary to ensure even and sufficient fixation of the tissue and muscle groups. The fixative solution consisted of 2% formaldehyde (Th. Geyer, Renningen, Germany), 30% ethanol, and 20% polyethylene glycol 400 (Th. Geyer, Renningen, Germany). Subsequently, the fixed limbs were stored overnight at +4 °C and then placed in a preservation bath consisting of a 2% formaldehyde solution for 8 weeks. Prior to preparation, the preserved forelimbs were soaked with preparation care solution (30% ethanol and 20% polyethylene glycol 400) and repeatedly moistened as needed during dissection. Between dissections, forelimbs were covered using cloths soaked in preparation care solution and stored in boxes at +4 °C. Dissections started by deskinning the canine forelimbs. Subsequently, muscles and nerves were dissected. To make underlying structures visible, the lateral head of the triceps brachii muscle and the superficial digital flexor muscle were cut at mid-length, angled approximately 90° to the muscle fiber orientation.

All specimens originated from animal cadavers that were obtained through the donation program of the Institute of Veterinary Anatomy of Freie Universität Berlin.

#### 2.2.2. Low-Fidelity 3D-Printed Models

**Figure 1:**
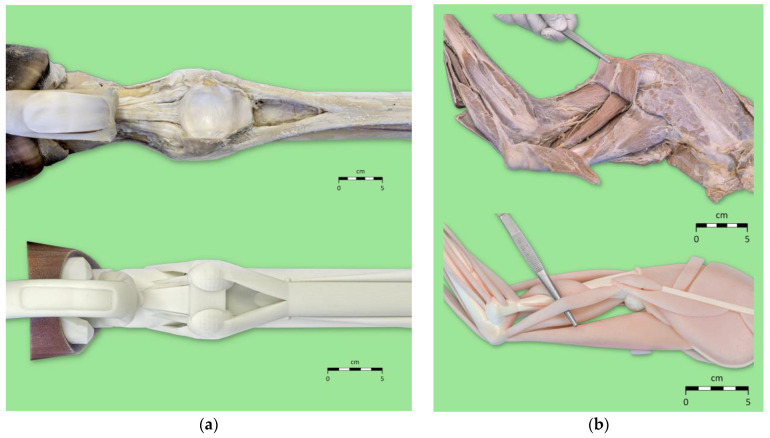
(**a**) Example for reduction of the equine distal limb ligaments and tendons in the low-fidelity 3D model (**below**) compared to the real specimen (**above**) by simplifying the shape of these structures, shown in palmar/plantar view. (**b**) Example of the reduction of the muscles in the low-fidelity 3D model of the canine forelimb (**below**) compared to the real specimen (**above**) by reducing the width and thickness of the muscles, shown in lateral view. In the real specimen, the tweezers lift part of the sectioned lateral head of the triceps brachii muscle. In the 3D model, the tweezers are placed under the lateral head of the triceps brachii muscle.

Low-fidelity 3D-printed models (3D models) of the equine distal limb and canine forelimb used in this study were modeled in the open-access software Blender 4.2.2 (Blender Foundation, Amsterdam, The Netherlands). Basic preset 3D models available within Blender were modified to represent the anatomical structures. In the sense of a low-fidelity model, the anatomical structures were displayed in a reduced form by minimizing the overall geometry of the structures, such as bones, muscles, and ligaments, while maintaining the correct positions of the structures as well as the correct origins and insertions of the muscles. Figure 1a shows an example of how the shape of the ligaments and tendons of the equine distal limb was simplified in the 3D model compared to the real specimen. Figure 1b shows a comparison of the lateral view of the canine forelimb of the real specimen and the 3D model. As part of the reduction of structures, the width and thickness of the muscles were reduced. As a result, for example, even without cutting the lateral head of the triceps brachii muscle, the other heads of this muscle, as well as other structures located below the lateral head of the triceps brachii muscle in the real specimen, are visible in the 3D model. Analogous to the real specimens, two models were made for the equine distal limb: a model representing the superficial structures (superficial view) and a model representing the deep structures (deep view). The anatomical structures of the canine forelimb were represented in one model. After the models were finalized in Blender, they were uploaded into the software GrabCAD Print 1.78 (Stratasys Ltd., Rehovot, Israel) to be prepared for 3D printing. After uploading, the software performed an automatic analysis and repaired detected issues where necessary. The models were subsequently positioned on the printing plate automatically. In GrabCAD Print, various model settings were selected for different anatomical structures, including color, shore value (degree of hardness), and surface finish (matt or glossy). Colors were defined using the RGBA (Red, Green, Blue, Alpha) color model, where values for red, green, and blue ranged from 0 to 255, and the alpha channel (A) controlled transparency on a scale from 0 (fully transparent) to 1 (fully opaque). The detailed main model settings can be found in Table 1. After selecting these model settings, the print time was automatically calculated and displayed.

For the equine distal limb, eight 3D models of the superficial view and eight 3D models of the deep view were printed. The length of the equine distal limb 3D models was 22 cm, with measurements taken from the proximal point of the cannon bone to the distal aspect of the hoof wall. Furthermore, 16 3D models of the canine forelimb were printed. The length of the 3D model of the canine forelimb was 31 cm, with measurements taken from the dorsal margin of the scapula to the coronary border of the third claw.

All models were printed using the Stratasys J55 multi-material 3D printer (Stratasys Ltd., Rehovot, Israel), which allows simultaneous printing of hard and soft materials using photopolymers. This capability enabled the production of certain parts of the model with mobility and flexibility; for example, the flexor tendons in the deep view model of the equine distal limb and the muscles in the canine forelimb model.

After printing, due to the liquid-based materials, the 3D models were completely encased in a water-soluble support material. The support material was removed by placing the printed 3D models in a water bath on a laboratory shaker (neoLab Multi Shaker Platt M, neoLab, Berlin, Germany) to facilitate the dissolution of the support material. Water was replaced periodically when it became cloudy. This process was carried out over several hours until most of the support material had dissolved. Any remaining residue of the support material was removed using a high-pressure water jet (Stratasys Ltd., Rehovot, Israel). Subsequently, the 3D models were dried and, if necessary, treated with baby or talcum powder to ensure that the material was free of any stickiness.

#### 2.2.3. Annotated Images of Real Specimens and 3D Models

**Figure 2:**
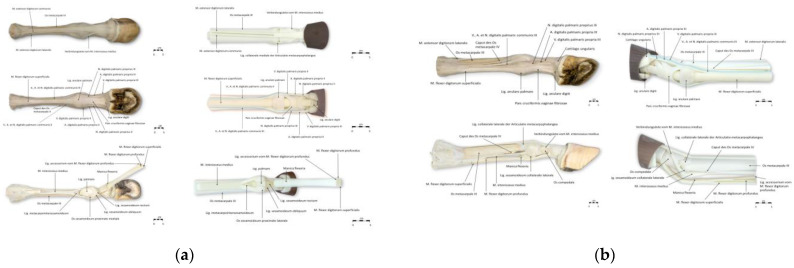
(**a**) Annotated images of a real specimen and a 3D model of the equine distal limb, shown in dorsal and palmar/plantar views. (**b**) Annotated images of a real specimen and a 3D model of the equine distal limb, shown in lateral view.

**Figure 3:**
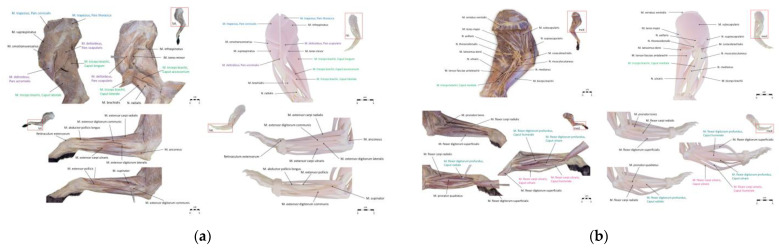
(**a**) Annotated images of real specimens and 3D models of the canine forelimb in lateral view. (**b**) Annotated images of real specimens and 3D models of the canine forelimb in medial view.

Images of the real specimens and the 3D models of the equine distal limb and the canine forelimb were created using a system camera (Sony, Alpha NEX-3, Nihonbashi, Tokyo, Japan). The images were edited using Adobe Photoshop (Version 22.0) and annotated in PowerPoint (Office PowerPoint 2019, Windows) using arrows. A total of 28 structures were annotated in the images of the equine distal limb. No distinction was made between thoracic and pelvic limbs, as only structures that are morphologically similar in the thoracic and pelvic limbs were labeled. For anatomical structures that differ in their nomenclature regarding palmar/plantar, palmar was used consistently in the annotation of the images. In the images of the canine forelimb, 45 structures were annotated. To aid in the identification of different parts of muscles, unique colors were used to mark different parts of the same muscle in the images of the canine forelimb. Structures that were not visible were not labeled. The annotated images of real specimens and 3D models are shown in Figure 2 and Figure 3.

### 2.3. Knowledge Assessment

**Figure 4:**
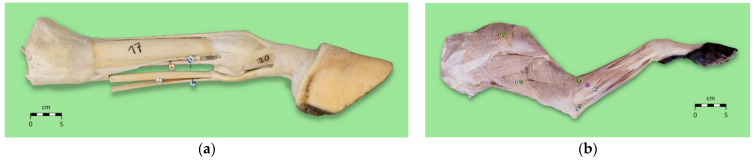
Real specimens of the equine distal limb (**a**) and canine forelimb (**b**) used for students’ knowledge assessment. Left, right, thoracic, and pelvic limbs were used as equine distal limb specimens. Both left and right forelimbs were used as canine forelimb specimens. Anatomical structures used in the knowledge assessment were labeled with numbers using sewn-on beads or a waterproof marker and are listed in Supplement S2.

At the end of each study, the learning outcomes were measured through a knowledge assessment in which students had to identify 20 anatomical structures on real specimens (equine distal limb in study 1, canine forelimb in study 2) and write down the corresponding anatomical terms on an examination sheet (Supplement S2). The knowledge assessment scores were defined as the learning outcomes. For the equine distal limb, both palmar and plantar were scored as correct in the knowledge assessment. In the specimens used in the knowledge assessment, anatomical structures were annotated using sewn-on beads labeled with numbers or a waterproof marker (Figure 4).

### 2.4. Study Design

Both studies were carried out during regular anatomy lessons in the dissection hall, which holds 32 tables for students to study in groups. To conduct the respective knowledge assessments immediately after the learning phase, the studies were carried out in two consecutive time slots, with half of the students present in each. This way, separate tables for the learning phase and the knowledge assessment could be prepared ahead of time. Study 1 focused on the equine distal limb, while study 2 focused on the canine forelimb.

#### 2.4.1. Study 1

All students were randomly assigned to one of two groups. One group received real specimens of the equine distal limb and the associated color-printed annotated images, while the other group learned with the 3D models of the equine distal limb and the associated color-printed annotated images. During the 10-min learning phase, students were divided into groups of 5–6 students per table and allowed to interact with each other. This phase was followed immediately by the test phase, in which students were asked to complete a knowledge assessment within 10 min. Each student completed the test individually. Before beginning the knowledge assessment, students indicated their assigned group (real specimen or 3D model) on their examination sheet. Prior to starting the knowledge assessment, the real specimens were covered with blankets. At the designated start time, all students simultaneously removed the blankets to access the annotated real specimens for the knowledge assessment. Students were asked to place their examination sheets face down on the table upon completion.

#### 2.4.2. Study 2

For study 2, students were randomly assigned to one of four groups. The real specimen group used real specimens of the canine forelimb, while the 3D model group worked with the 3D models of the canine forelimb. Additionally, two alternating groups were introduced: the 3D model/real specimen group, which first studied with the 3D models before switching to the real specimens, and the real specimen/3D model group, which started with the real specimens and then switched to the 3D models. As in study 1, students completed the learning phase at tables of 5–6 students each and were allowed to interact with each other during this time. In this study, the learning phase lasted 30 min for each group, with the two groups that worked with both learning materials in different orders switching materials after 15 min. During the learning phase, students were provided with color-printed annotated images matching their assigned learning materials. After the learning phase, all students completed the knowledge assessment individually. The knowledge assessment was carried out in the same way as in study 1. However, in this study, students were given 15 min for the knowledge assessment, to allow extra time to find all labeled structures, since some were partially hidden by other structures.

### 2.5. Student Evaluation

After each of the two studies, students were given access to the entire respective learning materials, i.e., real specimens and 3D models with the corresponding annotated images, to prepare for the regular exam, which was a practical exam on real specimens. Following the exam, participants were asked to complete a brief questionnaire about their learning experiences. The evaluation was conducted via LimeSurvey (Version 5.6.31). To assess the learning materials, either a six-point Likert scale (1 = strongly disagree, 6 = strongly agree) was used, or an open-ended question was answered. A detailed overview of the questions and response types is provided in Table 2 and Table 3.

### 2.6. Statistical Analysis

Statistical and descriptive analyses were performed using IBM SPSS Statistics Version 29^®^ (IBM, Armonk, NY, USA). Graphical representations were created using BioRender (©2025 BioRender, Toronto, ON, Canada). Each correct term in the knowledge assessment was awarded one point, while no points were given for incorrect or missing responses. Misspelled or incomplete terms were evaluated to determine whether they demonstrated recognition of the correct structure. To ensure reliability, two researchers independently assessed the test results. However, due to minimal deviations (<10% in both studies; κ = 0.94), the assessment by a single researcher was deemed sufficient for analysis. Prior to further analysis, data from both studies were visually inspected for normal distribution. Additionally, Levene’s test was used to test for homogeneity of variance. The significance level was set at 5%.

In study 1, the mean knowledge assessment score was calculated for the two study groups and compared using an unpaired *t*-test. Effect sizes were calculated using Cohen’s *d*. A d-value of 0.2 indicates a small effect, a d-value of 0.5 indicates a medium effect, and a d-value of 0.8 indicates a large effect [30]. In study 2, the mean knowledge assessment score was calculated for the four study groups and compared using a one-way analysis of variance (ANOVA). Bonferroni-corrected post-hoc analysis was used to verify significant differences between the study groups. Effect sizes were calculated using Cohen’s *f*, whereby an f-value of 0.1 indicates a small effect, an f-value of 0.25 indicates a medium effect, and an f-value of 0.84 indicates a large effect [30]. To assess the studies’ power, post-hoc power analyses were performed using G*Power 3.1.9.2.

The analysis of the Likert scale-based answers of the student evaluation was carried out for study 1 using an unpaired *t*-test (2 questions) and for study 2 using an ANOVA (4 questions). Raw data are provided in Supplement S3. A qualitative content analysis was carried out to assess the students’ responses to the open-ended questions in study 1 and study 2. For this purpose, the statements were paraphrased, then abstracted in the course of generalization, and finally pooled as codes in terms of reduction [31]. Coding categories can be found in Supplement S4. To ensure reliability, the codes generated were assigned to the students’ statements by a second researcher. However, given the minimal deviations (<10% in both evaluations; study 1: κ = 0.9; study 2: κ = 0.95), the assessment by a single researcher was considered adequate for the analysis.

## 3. Results

### 3.1. Number of Participants

In study 1, a total of 135 second-year veterinary students (71% of the enrolled students) participated, of whom 67 students were randomly assigned to the real specimen group and 68 were assigned to the 3D model group. In study 2, a total of 183 first-year veterinary students (88% of the enrolled students) participated. Of these, 44 students were randomly assigned to the real specimen group, 45 students to the 3D model group, 47 students to the 3D model/real specimen group, and 47 students to the real specimen/3D model group.

### 3.2. Learning Outcomes

#### Learning Outcomes in Study 1

**Figure 5:**
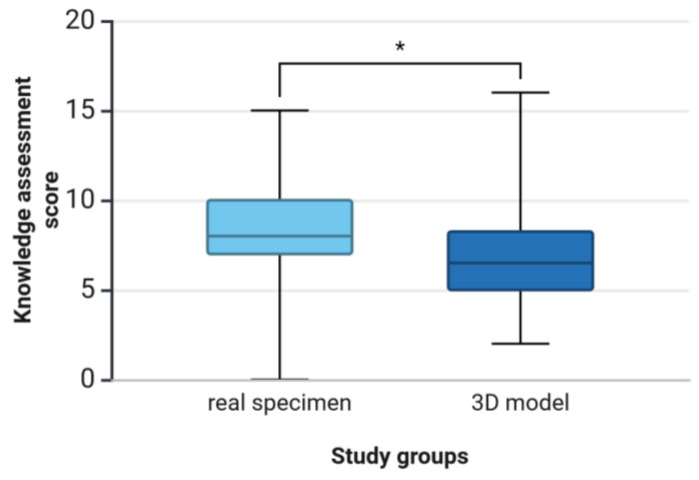
Study 1: Comparison of students’ knowledge assessment scores between study groups after learning anatomical structures of the equine distal limb for 10 min on either a real specimen (*n* = 67) or a 3D model (*n* = 68). The knowledge assessment was based on recognition of 20 anatomical structures labeled on a real specimen of the equine distal limb. Whiskers show 10th and 90th percentiles; * *p* < 0.05.

In study 1, the unpaired *t*-test revealed a significantly higher mean knowledge assessment score in the real specimen group (8.04 ± 3.14) compared to the 3D model group (6.91 ± 2.89) (*p* = 0.031) (Figure 5). Cohen’s *d* indicated a small to medium effect (*d* = 0.38). The post-hoc power analysis showed a statistical power of 0.99 (1-β err prob), which indicates sufficient power to detect the observed effect with a high degree of probability.

The structure that scored best in the knowledge assessment was the *Nervus digitalis palmaris proprius II/III* in the real specimen group (0.79 ± 0.41), while the best-scoring structure in the 3D model group was the *Musculus flexor digitorum superficialis* (0.78 ± 0.42). In both groups, the structure that scored lowest was the *Ligamentum metacarpointersesamoideum* (real specimen group: 0.13 ± 0.34; 3D model group: 0 ± 0). Raw data are provided in Supplement S3.

### 3.3. Learning Outcomes in Study 2

**Figure 6:**
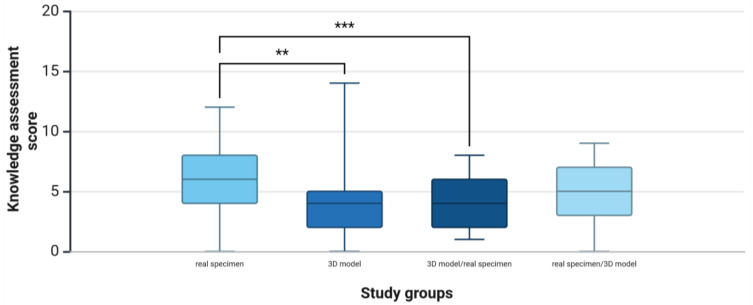
Study 2: Comparison of students’ knowledge assessment scores between study groups after learning anatomical structures of the canine forelimb for 30 min on a real specimen (*n* = 44), a 3D model (*n* = 45), a 3D model followed by a real specimen (3D model/real specimen, *n* = 47), or a real specimen followed by a 3D model (real specimen/3D model, *n* = 47). The knowledge assessment was based on recognition of 20 anatomical structures labeled on a real specimen of the canine forelimb. Whiskers show 10th and 90th percentiles; ** *p* < 0.01, *** *p* < 0.001.

The real specimen group achieved the highest mean score (6.11 ± 2.90), followed by the real specimen/3D model group (4.70 ± 2.43), the 3D model group (4.16 ± 2.90), and the 3D model/real specimen group (3.85 ± 1.93) (Figure 6). The study group had a significant influence on learning outcomes (*F*(6.87), *p* < 0.001, *ηp2* = 0.1, *n* = 183). The effect size indicates a medium effect (*f =* 0.33). The post-hoc power analysis showed a statistical power of 0.97 (1-β err prob), which indicates sufficient power to detect the observed effect with a high degree of probability. Post-hoc tests with Bonferroni correction revealed that not all study groups differed significantly. However, the mean knowledge assessment score in the real specimen group was significantly higher than the scores in the 3D model group (*p* = 0.002) and the 3D model/real specimen group (*p* < 0.001). 

The structure that scored best in the knowledge assessment for the real specimen group (0.66 ± 0.48), the 3D model group (0.58 ± 0.50), and real specimen/3D model group (0.62 ± 0.49) was the *Musculus biceps brachii*. For the 3D model/real specimen group (0.57 ± 0.50), it was the *Musculus serratus ventralis*. Structures with the lowest scores in the knowledge assessment were the *Musculus pronator teres* in the real specimen group (0 ± 0) and the *Musculus flexor carpi radialis* in the 3D model group (0 ± 0). In the 3D model/real specimen group, the *Musculus pronator teres* (0 ± 0) and the *Musculus coracobrachialis* (0 ± 0) scored equally low. In the real specimen/3D model group, the lowest scores were observed for the *Musculus pronator teres* and the *Musculus brachialis* (both 0.02 ± 0.15). Raw data are provided in Supplement S3.

### 3.4. Student Evaluation

#### 3.4.1. Student Evaluation in Study 1

**Figure 7:**
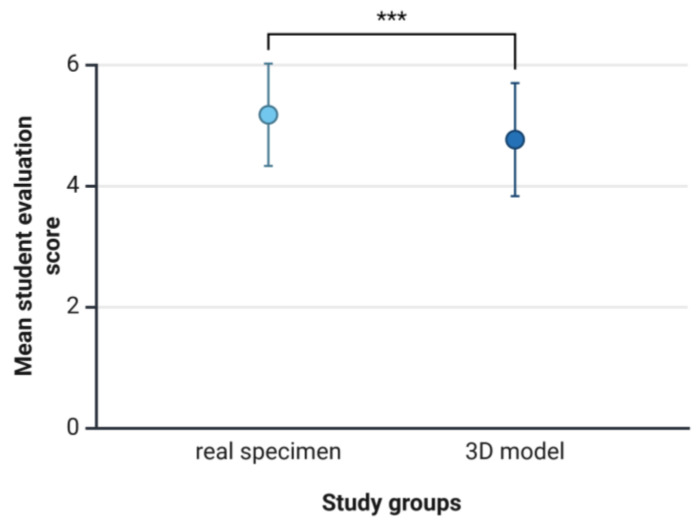
Study 1: Mean values and standard deviations of students’ evaluations (*n* = 159) of the learning materials. Based on a six-point Likert scale, students were asked to indicate whether they felt well prepared for a practical exam on a real anatomical specimen of an equine distal limb using the respective learning materials, i.e., real specimen or 3D model; *** *p* < 0.001.

Since all students had been given access to both the real specimens and the 3D models for exam preparation after study 1, 159 students took part in the evaluation of the learning materials after the exam (84% of the enrolled students). When asked which of the two learning materials students would prefer for exam preparation (assessment on a six-point Likert scale), the unpaired *t*-test revealed that students felt significantly better prepared for the practical exam by learning with the real specimens (5.17 ± 0.85) compared to the 3D models (4.76 ± 0.93) (*p* < 0.001) (Figure 7). Cohen’s *d* showed a medium effect (*d* = 0.47). The post-hoc power analysis showed a statistical power of 0.99 (1-β err prob), which indicates sufficient power to detect the observed effect with a high degree of probability. Raw data are provided in Supplement S3.

The qualitative content analysis of the answers to the open-ended question revealed that a high number of students (19 out of 57 statements; 33.3%) preferred the 3D model when starting to learn and then moved on to the real specimen. Other statements that were made more frequently indicated that the 3D models are better suited for gaining a general basic understanding of anatomical structures (eight out of 57 statements; 14.0%) and that it is a particular advantage that the 3D models can be used for learning outside the dissection hall (eight out of 57 statements; 14.0%). In addition, several students (seven of the 57 statements; 12.3%) expressed the wish to have further 3D models of other anatomical specimens available. The list of codes is provided in Supplement S4.

#### 3.4.2. Student Evaluation in Study 2

**Figure 8:**
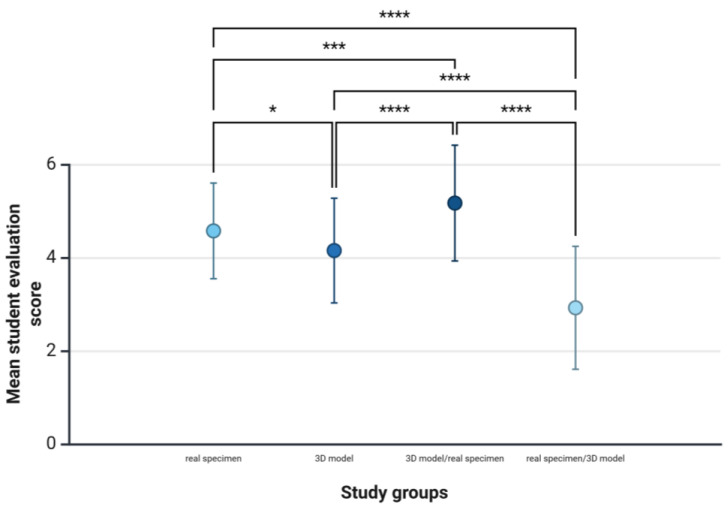
Study 2: Mean values and standard deviations of students’ evaluations (*n* = 180) of the learning materials. Based on a six-point Likert scale, students were asked to indicate whether they felt well prepared for a practical exam on a real anatomical specimen of a canine forelimb using the respective learning materials, i.e., real specimen, 3D model, 3D model followed by real specimen (3D model/real specimen), or real specimen followed by 3D model (real specimen/3D model); * *p* < 0.05, *** *p* < 0.001, **** *p* < 0.0001.

The evaluation after the regular exam, after all students had been given access to real specimens and 3D models for exam preparation, included 180 students in study 2 (87% of the enrolled students). Most of the students preferred the learning method 3D model/real specimen (5.17 ± 1.24), followed by the real specimen (4.57 ± 1.03), the 3D model (4.15 ± 1.12), and real specimen/3D model (2.92 ± 1.32) (Figure 8). The results of the student evaluation revealed that the preferred learning methods differed significantly (*F*(88.94), *p* < 0.0001, *ηp2* = 0.33, *n* = 180). The effect size indicates a strong effect (*f* = 0.7). The post-hoc power analysis showed a statistical power of 1.0 (1-β err prob), which indicates sufficient power to detect the observed effect with a high degree of probability. Post-hoc tests with Bonferroni correction showed that students felt significantly better prepared for the exam when using the learning materials in the order 3D model/real specimen compared to learning only with the real specimen (*p* < 0.001), only the 3D model (*p* < 0.0001), or when using the learning materials in the order real specimen/3D model (*p* < 0.0001). The differences in learning preference were also significant between real specimen and 3D model (*p* = 0.021), real specimen and real specimen/3D model (*p* < 0.0001), and 3D model and real specimen/3D model (*p* < 0.0001). The results of the evaluation show that students preferred the learning method that achieved the lowest learning outcomes in the knowledge assessment. Raw data are provided in Supplement S3.

The qualitative content analysis of students’ responses to the open-ended question revealed that almost half of the statements (17 out of 40 statements; 42.5%) indicated a preference for students to start learning with the 3D model and then continue with the real specimen. Furthermore, several statements indicated that students perceived learning with the 3D models as very helpful (six out of 40 statements; 15.0%). Some students indicated that they would prefer to learn with the 3D model and real specimen simultaneously (four out of 40 statements; 10.0%). The list of codes is provided in Supplement S4.

## 4. Discussion

The presented work investigated the impact of low-fidelity 3D-printed models as learning materials in veterinary anatomy classes. The research was guided by the hypothesis that students learning the anatomical structures of the equine distal limb or the canine forelimb using low-fidelity 3D-printed models would achieve similar learning outcomes as those using the corresponding real specimens.

When students learned with either the real specimen or the 3D model, as in study 1, students who learned with the real specimen achieved significantly higher learning outcomes than students who learned with the 3D model. In addition, the evaluation of the learning materials by students after the regular anatomy exam showed that students’ preference for using real specimens to prepare for the exam was significantly higher than for using the 3D model. To date, two studies on the use of 3D models as learning materials for veterinary anatomy have been published, both reporting similar findings as the present study [27,28]. A study from Japan demonstrated that 3D-printed skulls are effective as an introduction before working with real specimens [28]. Additionally, a study from Spain in the field of veterinary anatomy, which involved the creation of 3D models of various structures from various domestic and exotic species, including bone, viscera, and vascular structures, concluded that 3D-printed models are not a replacement for real specimens. Furthermore, the authors emphasized the need to improve 3D-printed models in terms of color, size, texture, and flexibility to enhance the anatomical accuracy and their potential application in surgical training [27]. It is possible that learning outcomes for 3D models differ depending on the structures and species used in the respective investigation. Since neither of those studies included the equine distal limb or the canine forelimb, comparability is limited.

Comparable results have been reported in studies on students of human medicine [32,33,34,35,36]. In a study carried out in human anatomy, students’ preferences for anatomy learning resources and their perceived effectiveness in achieving learning outcomes were investigated. Data collection took place at the end of the spring semester, during the abdominal gross anatomy course, after medical students had completed the upper and lower limb sections, as well as the cardiovascular and respiratory system modules. The results showed that real specimens combined with clinical tutorials were rated as the preferred and most effective resource for learning anatomy, followed by dissection videos, other electronic resources, and printed materials. In contrast, the perceived usefulness of plastinated specimens and plastic models was rated lower by students [32]. Other studies in the field of human anatomy also confirm that both students and teachers rate the impact of real specimens as learning materials higher than that of 3D models [33,34]. Moreover, there is no substantial evidence that 3D models are significantly more effective in enhancing learning outcomes compared to real specimens [33,34]. In studies in which students were asked about their learning experience with 3D-printed models of human anatomical specimens compared to plastinates, students found plastinates superior to 3D models due to their more realistic nature. However, students emphasized that they would prefer 3D-printed models for learning basic anatomy [35,36]. Overall, students perceived the usefulness of 3D-printed models, particularly in gaining a general, basic understanding of anatomical structures, while real specimens or plastinates might be more likely to be used to deepen knowledge and understanding in a practical context [34,35]. In the study of Mogali et al., 3D-printed models of the thoracic limb were evaluated [35], while cardiac, head, and neck 3D-printed models were tested by Radzi et al. [36]. The student evaluations of learning materials in those studies showed similar results to the student evaluation in the present study [35,36]. Thus, in study 1, many students stated that the 3D models might be better suited than the real specimens for initially gaining a general, basic understanding of anatomical structures. Therefore, in addition to the two test groups that used the real specimens or the 3D models for learning, two further test groups were set up in study 2, which used both learning materials, real specimens and 3D models, in different orders. However, results from study 2 show that the group that had learned with the real specimens had the highest learning outcomes, followed by the real specimen/3D model group and then the 3D model group. The group with the lowest learning outcomes was the 3D model/real specimen group, which was the group that had used the learning materials in the order that, according to the students’ evaluation, should have been the most suitable. As in study 1, many students who participated in study 2 also stated in the open-ended question that they would consider it the best learning method to start with the 3D-printed models and then transition to the real specimens. The analysis of quantitative data from students’ evaluations also showed that students regarded learning in the order 3D model/real specimen as most suitable, followed by learning with only the real specimen and then the 3D model only. Learning in the order real specimen/3D model was found to be least suitable by students. Students’ preference for starting anatomy learning with a 3D model before transitioning to the real specimen can be explained on the basis that students often experience considerable apprehension, stress, and anxiety when encountering cadavers [37,38,39]. In this context, 3D models may serve as an effective introductory tool, helping students become familiar with anatomical structures, easing their discomfort, and reducing psychological barriers to working with cadavers, which remain a vital component of the anatomy curriculum [40]. In clinical training, 3D models can ease the transition to working with live animals by reducing student stress and fostering greater interest in clinical practice, for example, when they learn clinical skills such as intravenous catheterization, bandaging, or anesthesia on canine 3D-printed models [9]. The results show that students’ perception of which learning method would lead to the highest learning outcomes did not match the learning method that ultimately led to the highest learning outcomes. This finding is consistent with the results of several studies that have identified a discrepancy between the learning outcomes perceived by students and the learning outcomes they actually achieved [41,42,43]. This could be the case because students might be more likely to perceive an increase in self-efficacy rather than measuring the actual acquisition of skills [41]. However, the lower learning outcomes when learning in the order 3D model/real specimen observed in the presented study could also arise due to students requiring more time to become familiar with the 3D models, as they were using a new and therefore unfamiliar learning tool, necessitating additional time to adapt [2,18]. In a study that evaluated a low-fidelity inguinal canal model, students explicitly expressed a desire for more hands-on time with the model [18]. Similarly, the findings of a previous study on the use of 3D scans as learning materials emphasized the vital importance of giving students sufficient time to adjust to new learning circumstances [44]. Thus, students who started learning with the 3D models may struggle to adapt and apply their knowledge effectively when switching to the real specimens. This aspect could have been underestimated by students in their evaluation. Overall, it must be considered that students’ preferences are not necessarily a reliable indicator of learning efficacy, as shown in the studies presented, as well as in previous studies [32,33,34,35,36].

In the learning material evaluation, some students indicated that they would prefer to learn with the real specimens and the 3D models simultaneously. This way, the advantages and disadvantages of both learning materials could possibly counterbalance each other. Since dissection alone cannot meet all educational needs, combining it with innovative teaching methods offers a comprehensive approach to anatomy education, ensuring both tradition and technology work together to enhance learning [45].

An advantage of the 3D models highlighted by students’ evaluations is that they can effectively support learning beyond the dissection hall. Thus, the use of 3D-printed models expands access to anatomical specimens, helping to mitigate the challenges posed by limited cadaver availability [2,28,46,47].

Not least, students expressed enthusiasm for the 3D-printed models as they perceived the 3D-printed models as highly beneficial for learning. This aligns with previous research, which has similarly reported that students find 3D-printed models to be valuable educational tools [2,9,12,13,14,18,19,48]. Accordingly, several students indicated that they wish to have further 3D-printed models of other anatomical specimens available. This positive commitment should be preserved, as it enhances students’ motivation to engage in anatomy learning. At this point, it is important to emphasize that 3D models are tools that can be useful but are not replacements in anatomy education [27,28,35,36].

Some limitations may affect the interpretation and transferability of the results. First, the studies had only one assessment point. Further research could examine whether the same results would be obtained if there were a second assessment point with a longer time interval after the study. Second, both anatomical specimens addressed in the studies were from the musculoskeletal system. Further studies could clarify the extent to which low-fidelity 3D-printed models of specimens from other organ systems can lead to comparable learning outcomes to those of the corresponding real specimens. Third, the tests in the studies focused only on the recognition of anatomical structures on the specimen. The fact that students frequently stated that they found the 3D models helpful for initial learning could also indicate that the 3D models may be more suitable for understanding functional aspects and relationships, for example, in the muscles and ligaments. In further studies, these aspects should be considered in the tests to verify this assumption. Fourth, it is assumed that if more time were available, transitioning between different learning resources might be beneficial [45]. To examine whether a period to familiarize with the 3D model or the real specimen could reduce the differences in the learning outcomes in those groups that change the learning material during the learning period, such a period should be introduced for those test groups in subsequent studies. Lastly, this study explored the use of low-fidelity models in veterinary anatomy, and it may be possible that better learning outcomes could be achieved with high-fidelity models.

## 5. Conclusions

The studies did not confirm the hypothesis that the same learning outcomes can be achieved with low-fidelity 3D-printed models of the equine distal limb or the canine forelimb as with the corresponding real specimens. For both the canine forelimb and the equine distal limb, participants who studied using real specimens achieved significantly higher learning outcomes compared to those who used 3D models. In contrast, students stated in the evaluation that they would prefer to start learning with the 3D models and then switch to the real specimens. However, in the study, students achieved the lowest learning outcomes with this learning method. This discrepancy in the students’ evaluation of learning materials could be, on the one hand, because the students might be more likely to measure the increase in self-efficacy than the increase in knowledge. On the other hand, it could be because familiarizing oneself with a new learning material takes some time, an aspect that was neglected in the study, which used the same learning time for all test groups, resulting in a potential disadvantage for the groups that used both learning materials in alternating order. In conclusion, equine distal limb and canine forelimb 3D models appear to be less effective for knowledge acquisition. While they may serve as useful preparatory tools, they should not be considered substitutes.

Beyond that, students were enthusiastic about the 3D models of the equine distal limb and the canine forelimb and expressed the wish to have more 3D models of further anatomical specimens, which showed that the 3D models could increase students’ learning motivation. In addition, the 3D models expand opportunities for students to learn anatomy from specimens, since they can also be used outside of the dissection hall.

## Figures and Tables

**Table 1:** Main model settings in the GrabCAD Print software, including color using the RGBA color model (range 0 to 255), hardness using Shore-A (range 45 to 100) and the characteristic of the superficial surface using Surface Finish (matt/shiny).

Anatomical Structures	Color	Shore-A	Surface Finish
Bones	255(R) 253(G) 230(B) 1(A)	off	matt
Muscles	252(R) 200(G) 209(B) 0.18(A)	50	matt
Tendons	255(R) 253(G) 230(B) 0.18(A)	50	matt
Nerves	242(R) 235(G) 190(B) 0.56(A)	70	matt
Arteries	242(R) 235(G) 190(B) 0.56(A)	70	matt
Veins	108(R) 185(G) 207(B) 0.41(A)	70	matt
Hoof	107(R) 62(G) 3(B) 1 (A)	off	matt

**Table 2:** Questions and response types of the student evaluation in study 1.

Question	Response Type
By learning with the real specimen, I felt well prepared for a practical exam on an anatomical specimen.	Likert scale
2. By learning with the low-fidelity 3D-printed model, I felt well prepared for a practical exam on an anatomical specimen.	Likert scale
3. Do you have any feedback for us regarding the low-fidelity 3D-printed models?	Free text

**Table 3:** Questions and response types of the student evaluation in study 2.

Question	Response Type
1. By learning with the real specimen, I felt well prepared for a practical exam on an anatomical specimen.	Likert scale
2. By learning with the low-fidelity 3D-printed model, I felt well prepared for a practical exam on an anatomical specimen.	Likert scale
3. I think it makes sense to first learn with the low-fidelity 3D-printed model and then with the real specimen for a practical examination on an anatomical specimen.	Likert scale
4. I think it makes sense to first learn with the real specimen and then with the low-fidelity 3D-printed model for a practical examination on an anatomical specimen.	Likert scale
5. Do you have any feedback for us regarding the low-fidelity 3D-printed models?	Free text

## Data Availability

Data are contained within the article or Supplementary Material. The data presented in this study are available in the Supplementary Material.

## Supplementary Materials

The following supporting information can be downloaded at https://www.mdpi.com/2076-2615/15/10/1380/s1, Supplement S1: Information letter and data protection declaration; Supplement S2: Examination sheets; Supplement S3: Raw data; Supplement S4: List of codes for qualitative content analysis.
