# Supplementary material for: The Impact of Low-Fidelity Three-Dimensional-Printed Models of the Equine Distal Limb and the Canine Forelimb in Teaching Veterinary Anatomy in Practical Classes
Source: Animals (Basel). 2025 May 10;15(10):1380. doi: 10.3390/ani15101380 (PMC12108517; doi:10.3390/ani15101380)
Supplement: Supplementary file 1 [file animals-15-01380-s001.zip › Supplement S1.pdf]

“Investigation of the didactic potential of printed 3D models in veterinary anatomy teaching”

Dear students of veterinary medicine,

Thank you for agreeing to take part in the study “Investigation of the didactic potential of 3D printed models in veterinary anatomy teaching”. We would like to inform you in advance about the study, the procedure and some data protection aspects. Please read the following information carefully.

OBJECTIVE OF THE STUDY: This study investigates the added value of 3D printed models as a learning medium in veterinary anatomy teaching. The study is solely for scientific purposes and to improve the quality of teaching and is part of a dissertation project.

WHAT YOUR PARTICIPATION CONSISTED OF: You will receive either the native anatomical specimen (control group) or printed 3D models of the anatomical specimen (test group) or both (study 2) for the topics regularly covered in the anatomy course. In order to measure learning success, you will be given an anonymized knowledge test at the end of the study and a voluntary evaluation of the learning materials at a later date.

TIME FRAMEWORK/EXPENSES: The study will be carried out as part of the anatomy course using the topics regularly covered in the course, so that you will not have any additional time or learning effort.

RISKS: Following the study, all learning materials will be made available to all students, so that you will not suffer any disadvantages regardless of whether you are in the control or test group.

BENEFITS: Participation in the study offers you the opportunity to contribute to the development of new and innovative teaching methods in veterinary anatomy.

CONFIDENTIALITY AND DATA PROTECTION: All data will be collected anonymously. The declarations of consent are stored in a separate folder in a secure location accessible only to the project management.

The data is protected in accordance with the strictest data protection regulations in accordance with the European General Data Protection Regulation (GDPR), which came into force on 25 May 2018. The survey data will only be processed by the persons involved in the project and will only be used for the purposes of research and quality assurance of studies and teaching.

CONSENT TO PARTICIPATE AND WITHDRAWAL: Participation in the study is completely voluntary and you will not suffer any disadvantages if you do not participate. You have the option to withdraw from the study at any time by informing us that you no longer wish to participate in the study. This will also not result in any disadvantages for you and no questions will be asked about the reasons for your withdrawal.

Data protection declaration of consent: I hereby declare that I have carefully read and understood the above information and explanations. I give my consent to the collection, storage, processing and use of my data solely for the purpose of answering scientific questions in connection with this study.

First name, last name | Date | Signature
