# Supplementary material for: The Impact of Low-Fidelity Three-Dimensional-Printed Models of the Equine Distal Limb and the Canine Forelimb in Teaching Veterinary Anatomy in Practical Classes
Source: Animals (Basel). 2025 May 10;15(10):1380. doi: 10.3390/ani15101380 (PMC12108517; doi:10.3390/ani15101380)
Supplement: Supplementary file 1 [file animals-15-01380-s001.zip › Supplement S2.pdf]

Please select group: **3D model** ☐ **native specimen** ☐

Test

	Name of the structure
1	*Correct answer: Vena digitalis palmaris propria II/ III*
2	*Correct answer: Arteria digitalis palmaris propria II/ III*
3	*Correct answer: Nervus digitalis proprius II/ III*
4	*Correct answer: Manica flexoria*
5	*Correct answer: Caput of the Metacarpus II/ IV*
6	*Correct answer: Ligamentum metacarpointersesamoideum*
7	*Correct answer: Ligamentum sesamoideum obliquum*
8	*Correct answer: Ligamentum rectum*
9	*Correct answer: Connecting branches of the Musculus interosseus medius*
10	*Correct answer: Musculus interosseus medius*
11	*Correct answer: Cartilago ungularis*
12	*Correct answer: M. flexor digitorum [digitalis] superficialis*
13	*Correct answer: Musculus flexor digitorum [digitalis] profundus*
14	*Correct answer: Ligamentum anulare palmare*
15	*Correct answer: Pars cruciformis vaginae fibrosae*
16	*Correct answer: Ligamentum anulare digiti*
17	*Correct answer: Metacarpus III*
18	*Correct answer: Musculus extensor digitorum [digitalis] lateralis*
19	*Correct answer: Musculus extensor digitorum [digitalis] communis*
20	*Correct answer: Os compedale*

Please select group: **native specimen** ☐

3D model ☐

3D model/ native specimen ☐

native specimen/ 3D model ☐

Test

	Name of the structure
1	*Correct answer: Musculus extensor digitorum [digitalis] lateralis*
2	*Correct answer: Musculus extensor digitorum [digitalis] communis*
3	*Correct answer: Musculus extensor carpi radialis*
4	*Correct answer: Musculus pronator teres*
5	*Correct answer: Musculus flexor carpi radialis*
6	*Correct answer: Nervus ulnaris*
7	*Correct answer: Nervus radialis*
8	*Correct answer: Nervus suprascapularis*
9	*Correct answer: Musculus coracobrachialis*
10	*Correct answer: Musculus anconeus*
11	*Correct answer: Musculus infraspinatus*
12	*Correct answer: Musculus omotransversarius*
13	*Correct answer: Musculus serratus ventralis*
14	*Correct answer: Musculus triceps brachii, Caput longum*
15	*Correct answer: Musculus biceps brachii*
16	*Correct answer: Musculus supraspinatus*
17	*Correct answer: Musculus brachialis*
18	*Correct answer: Musculus subscapularis*
19	*Correct answer: Musculus teres major*
20	*Correct answer: Musculus latissimus dorsi*
